# DEMENTIA CARE PARTNER ACTIVATION PROGRAM FOR FILIPINO AMERICANS

**DOI:** 10.1093/geroni/igad104.3262

**Published:** 2023-12-21

**Authors:** Mary Dioise Ramos, Nazmus Sakib, Mehedi Hasan

**Affiliations:** Kennesaw State University, Kennesaw, Georgia, United States; Kennesaw State University, Kennesaw, Georgia, United States; Kennesaw State University, Kennesaw, Georgia, United States

## Abstract

This study aims to enhance the quality of life and health outcomes for individuals with Alzheimer’s Disease and Related Dementia (ADRD) and their care partners through a culturally tailored support program. Specifically focusing on the unique challenges Filipino and Filipino American (FA) caregivers face, we seek to address disparities in access to care and develop strategies for effective health system navigation. The research employs a multifaceted approach, combining surveys, focus groups, observational techniques, and interviews to assess the caregiving experience comprehensively. Our primary objectives include evaluating the impact of caregiving burden, socio-emotional support, and health literacy on emergency department visits and hospital admissions for ADRD patients. Preliminary results highlight the significance of culturally sensitive interventions. FA caregivers often encounter difficulties due to cultural beliefs, knowledge gaps about ADRD, and limited support networks. This study outlines the development and testing of a specialized program designed to empower FA caregivers with the skills needed for proficient care management and emergency response. By engaging these caregivers in participatory action research, the program aims to foster active involvement and enhance their caregiving capabilities. The study’s outcomes emphasize the potential for improved quality of life and health outcomes for individuals with ADRD and their caregivers. By addressing FA caregivers’ unique challenges in the context of disparity populations, our research contributes to enhancing health system navigation behaviors and promoting culturally attuned care practices. This initiative promises to reduce emergency department visits and hospital admissions, benefiting ADRD patients and their dedicated care partners.

